# Analysis of the effect of CYP2C19 gene properties on the anti-platelet aggregation of clopidogrel after carotid artery stenting under network pharmacology

**DOI:** 10.1186/s40360-024-00750-w

**Published:** 2024-06-06

**Authors:** Pengfei Li, Mengying Cao, Ling Liu, Long Chen, Shuang Liang, Youbin Wang

**Affiliations:** 1 Interventional Radiology Department, Xuzhou Cancer Hospital, Xuzhou, 221000 China; 2 Pharmacy Department, Xuzhou Central Hospital, Xuzhou, 221009 China

**Keywords:** Network pharmacology, CYP2C19 gene polymorphism, CAS, Clopidogrel, Antiplatelet

## Abstract

Antiplatelet therapy is an important factor influencing the postterm patency rate of carotid artery stenting (CAS). Clopidogrel is a platelet aggregation inhibitor mediated by the adenosine diphosphate receptor and is affected by CYP2C19 gene polymorphisms in vivo. When the CYP2C19 gene has a nonfunctional mutation, the activity of the encoded enzyme will be weakened or lost, which directly affects the metabolism of clopidogrel and ultimately weakens its antiplatelet aggregation ability. Therefore, based on network pharmacology, analyzing the influence of CYP2C19 gene polymorphisms on the antiplatelet therapeutic effect of clopidogrel after CAS is highly important for the formulation of individualized clinical drug regimens. The effect of the CYP2C19 gene polymorphism on the antiplatelet aggregation of clopidogrel after CAS was analyzed based on network pharmacology. A total of 100 patients with ischemic cerebrovascular disease who were confirmed by the neurology department and required CAS treatment were studied. CYP2C19 genotyping was performed on all patients via a gene chip. All patients were classified into the wild-type (WT) group (*1/*1), heterozygous mutation (HTM) group (CYP2C19*1/*2, CYP2C19*1/*3), and homozygous mutation (HMM) group (CYP2C19*2/*2, CYP2C19*2/*3, and CYP2C19*3/*3). High-performance liquid chromatography (HPLC) with tandem mass spectrometry (MS/MS) was used to detect the blood concentration of clopidogrel and the plasma clopidogrel clearance (CL) rate in different groups of patients before and after clopidogrel treatment. The platelet aggregation rate of patients with different genotypes was measured by turbidimetry. The incidences of clopidogrel resistance (CR) and stent thrombosis in different groups after three months of treatment were analyzed. The results showed that among the different CYP2C19 genotypes, patients from the HTM group accounted for the most patients, while patients from the HTM group accounted for the least patients. Similarly, the clopidogrel CL of patients in the HMM group was lower than that of patients in the WT group and HTM group (*P* < 0.01). The platelet inhibition rate of patients in the HMM group was evidently inferior to that of patients in the WT group and HTM group (*P* < 0.01). The incidence of CR and stent thrombosis in the WT group was notably lower than that in the HTM and HMM groups (*P* < 0.01). These results indicate that the CYP2C19 gene can affect CR occurrence and stent thrombosis after CAS by influencing clopidogrel metabolism and platelet count.

## Introduction

Cerebrovascular disease is common in elderly individuals. In recent years, with the acceleration of aging in China, the incidence of cerebrovascular disease has shown a dramatic upward trend, and cerebrovascular disease has become one of the main causes of death in elderly people [1]. According to statistics, the annual increase in the prevalence of cerebrovascular diseases in China is more than 5% [2], and ischemic cerebrovascular diseases caused by carotid artery stenosis account for 25% of cerebrovascular diseases [3]. Carotid artery stenting (CAS) is widely adopted in the treatment of ischemic cerebrovascular diseases caused by carotid artery stenosis due to its advantages of rapid recovery, short operation time, and low incidence of adverse events. However, CAS, an invasive surgical method, has a certain impact on the vascular function of patients, and the incidence of postoperative restenosis is as high as 5-20% [4]. Postoperative restenosis refers to successful interventional surgery involving the treatment of local coronary damage after the “healing” reaction caused by local vascular lumen restenosis. Postoperative antiplatelet therapy is an effective method for preventing postoperative restenosis after CAS and is also an important factor for the long-term patency rate after CAS [5]. Antiplatelet therapy with clopidogrel after CAS can effectively reduce the incidence of restenosis and thrombosis after CAS [6].

Clopidogrel, a commonly used antiplatelet aggregation drug, has no antiplatelet activity by itself and can exert its antiplatelet effect only after the action of the cytochrome P450 (CYP) enzyme system. As an important member of the P450 (CYP) enzyme system, CYP2C19 plays an important role in the metabolism of multiple drugs. Relevant research has shown that CYP2C19 is the ring-limiting enzyme of clopidogrel in liver cell metabolism and plays an important role in clopidogrel’s antiplatelet activity [7], and the CYP2C19 gene is highly polymorphic. The wild-type strain (*1/*1) is the normal genotype, while the enzyme activity encoded by the CYP2C19*2 and CYP2C19*3 mutants is weakened or even lost, which seriously affects the metabolism and activity of clopidogrel, leading to the weakening or inhibition of its antiplatelet aggregation ability [8]. Clinical research has shown that approximately 1/4 of patients develop clopidogrel resistance after treatment with clopidogrel [9]. The probability of mutation of the CYP2C19 gene in the Asian population is up to 30% [10]; therefore, the incidence of clopidogrel resistance in the Asian population is greatly superior to that in the white population [11]. Many clinical studies have suggested that the incidence of adverse events after percutaneous coronary intervention in patients with CYP2C19 gene mutations is dramatically greater than that in wild-type patients. Moreover, ischemic stroke patients with CYP2C19 gene mutations have a poor response to clopidogrel and poor prognosis [12, 13]. However, the effect of the CYP2C19 gene polymorphism on the antiplatelet function of clopidogrel after CAS is still unclear.

Therefore, in this study, CYP2C19 genotyping of patients with ischemic cerebrovascular disease was performed using gene chip technology, and the influence of CYP2C19 gene polymorphisms on the antiplatelet therapeutic effect of clopidogrel after CAS was analyzed based on network pharmacology, providing a reference basis for the formulation of individualized clinical medications.

## Materials and methods

### Research object and grouping

The data from this study were collected from 100 patients who were diagnosed with ischemic cerebrovascular disease in the Department of Cardiology, Our Hospital, from December 2020 to March 2022 and who required CAS treatment. There were 66 males and 34 females, ranging in age from 51 to 83 years, with an average age of 65.63 ± 9.21 years. All patients were classified into a wild-type (WT) group (*1/*1), a heterozygous mutation (HTM) group (CYP2C19*1/*2, CYP2C19*1/*3), and a homozygous mutation (HMM) group (CYP2C19*2/*2, CYP2C19*2/*3, and CYP2C19*3/*3).

The inclusion criteria for patients were as follows: (i) had a diagnosis of ischemic cerebrovascular disease, (ii) had a CAS-accepted status, and (iii) received clopidogrel antiplatelet therapy. The exclusion criteria for patients were as follows: (i) had cerebral embolism, (ii) had contraindications for antiplatelet therapy, (iii) had abnormal coagulation function, (iv) had severe liver disease, (v) received clopidogrel within three months before surgery, and (vi) had a history of intracranial hemorrhage. The experimental procedure of this study was approved by the Ethics Committee of Xuzhou Cancer Hospital, and all the subjects included in the study signed informed consent forms.

### CAS and postoperative antiplatelet therapy

The patient was placed in the supine position, routine disinfection was performed under general anesthesia, and a 5F125 cm angiography catheter was introduced. Angiography was performed to determine the location of the diseased vessels and measure their diameter. A spider (EV3 Corporation) and 0.014 Transcnd Platinum microguide wires (Stryker Corporation) were used. The Spider was guided 5 cm distal to the stenosis, and the Transend Platinum guidewire was withdrawn. A balloon of appropriate size was introduced to dilate the diseased vessels. The balloon was withdrawn after maintaining working pressure for approximately 30 s, after which the stent was introduced to the diseased vessels. Angiography was performed again to confirm that the stent was dilated and in good position. The doctor removed the catheter sheath, wrapped up the puncture wound, and ended the treatment. After surgery, the patient was instructed to immobilize the right lower limb for 12 h and fast for 6 h, and corresponding postoperative care was given. All patients received aspirin enteric-coated tablets (100 mg/day, Bayer Healthcare Co., Ltd., National Drug Approval J20130078) and clopidogrel tablets (75 mg/day, Sanofi (Hangzhou) Pharmaceutical Co., Ltd., National Drug Approval J20130083) 3 days before surgery and 3 months after surgery.

### Pharmacokinetics of clopidogrel

Blood (2 mL) was collected from all patients for pharmacokinetic analysis of clopidogrel before and 8 h after medication. Blood was collected and centrifuged at 3,000 r/min at 4 °C for 10 min. Plasma was collected, 100 µL of plasma was added to 50 µL of ticlopidine at a concentration of 100 ng/mL, mixed and added to 50 µL of NaOH at a concentration of 1 mol/L. Then, 3 mL of n-hexane: ether (1:4) solution was added, and the mixture was shaken and centrifuged at 3,500 r/min for 5 min. Reference [14] employed liquid chromatography‒mass spectrometry-mass spectrometry (LC‒MS-MS) for detecting clopidogrel concentrations in plasma. The specific procedure involved the collection of the supernatant, which was dissolved in the mobile phase. Subsequently, 20 µL was subjected to LC‒MS-MS analysis using a C18 column. The mobile phase consisted of methanol-10 mmol/L ammonium acetate solution (85:15), and the column temperature was 25 °C. For LC‒MS-MS analysis, the injection volume was 10 µL, the flow rate was 110 mL/min, and a shunt ratio of 9:1 was utilized. To prepare quality control samples at concentrations of 0.2 ng/mL, 5 ng/mL, and 20 ng/mL, 100 µL of blank plasma was mixed with varying volumes of clopidogrel solution (0.5 mg/mL). Six sample analyses were performed for each concentration over three consecutive days. The concentrations of the samples were calculated based on the standard curve to assess the intraday and interday precision (denoted as relative standard deviation (RSD)) and accuracy (denoted as relative error (RE)). The accuracy and precision of the LC‒MS‒MS method were evaluated based on the determination of the quality control samples.

The concentration of clopidogrel at different time points before medication (0 h) and 8 h after medication was detected according to the analysis results, and the plasma clearance rate (CL) [15, 16] of clopidogrel was calculated as follows.

1 $$C L=\frac{C_0-C_8}{\Delta t}$$

**Fig. 1:**
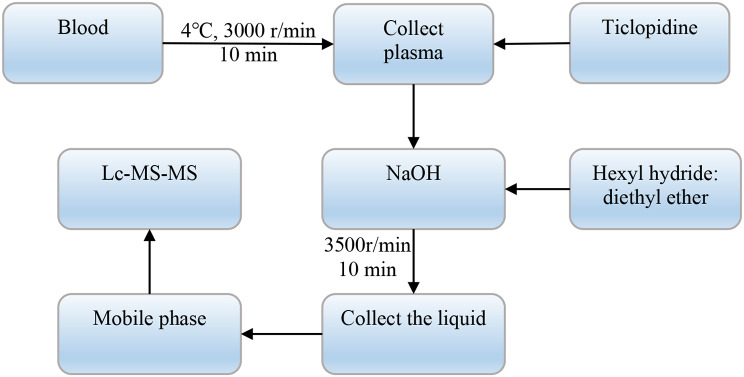
Flow chart of the pharmacokinetic analysis of clopidogrel

*C*_0_ and *C*_8_ represent the clopidogrel concentrations detected in the blood of patients before and 8 h after medication, respectively, and ∆*t* refers to the time interval of medication. The specific pharmacokinetic analysis process of clopidogrel is shown in Fig. 1.

Different volumes of clopidogrel solution (0.5 mg/mL) were added to 100 µL of plasma to achieve final clopidogrel concentrations of 0.1 ng/mL, 0.5 ng/mL, 1.0 ng/mL, 2.0 ng/mL, 5.0 ng/mL, and 20 ng/mL. Samples with different concentrations were prepared for 0 h and 12 h at room temperature (25 °C), 24 h at room temperature, 12 h at -20 °C, 24 h at -20 °C, 30 d at -20 °C, and 60 d at -20 °C for sample stability testing.

### Genotyping of CYP2C19 via gene chip technology

The whole-blood DNA Extraction Kit (catalog number: K182104A; Invitrogen) was used for total DNA extraction, which was performed as follows. A total of 300 µL of cell lysis buffer was added to 300 µL of whole blood and mixed thoroughly, followed by incubation at room temperature for 10 minutes. Centrifugation was performed at 13,000 r/min for 1 min, after which the precipitate was collected. The nuclear lysate was added to suspend the precipitate, 1.5 µL of RNA was added, and the mixture was reversed and mixed gently. The mixture was then left for 15 min at 37°C and centrifuged for 3 min at 13,000 r/min, after which the supernatant was collected. Isopropanol was added, and the mixture was centrifuged at 13,000 r/min at room temperature for 1 min to collect the precipitate. The primer sequences for CYP2C19 gene amplification were as follows: w1-F: 5’-TGTGCTCCCTGCAATGTGAT-3’; w1-R: 5’-ATGGAGTGAT ATAAGCACGCTTTG-3’. Then, 100 µL of the DNA precipitate was added, and the mixture was stored in a refrigerator at 4 °C. The YP2C19 gene was amplified by a PCR apparatus (Light Cycler 480, Roche) using a 25 µL CYP2C19 amplification system and annealed at 50 °C for 30 s. The DNA was removed, and the CYP2C19 gene was hybridized and genotyped with an automatic nucleic acid molecular hybridization instrument (BR-526-24, Shanghai Baiao Technology Co., Ltd., China). After the hybridization reaction, the chip was removed, dried at room temperature, and then put into a BE-2.0 biochip reader (Shanghai Baiao Technology Co., Ltd., China) for detection, after which the results were recorded.

### Pharmacodynamic analysis of clopidogrel

Two milliliters of venous blood was collected and added to an EDTA anticoagulant tube, which was divided into two parts. Then, 300 µL of sodium citrate (3.8%) was added to some of the samples, which were subsequently centrifuged at 1,000 r/min for 10 min. The upper plasma was collected to prepare platelet plasma (PRP), and the remaining blood was centrifuged at 3,000 r/min for 20 min to collect the upper plasma to prepare platelet-poor plasma (PPP). In reference [17], the turbidimetric method was adopted to detect platelet aggregation, and modifications were made on this basis. PRP and PPP were counted under an optical microscope, and their concentrations were adjusted to 200 × 109/L. Then, 500 µL PPP was added to a turbidimetry tube under a whole-blood aggregator (PACK-4, Helena), and the transmittance was adjusted to 100% at a wavelength of 620 nm. Then, 500 µL of PRP was added to the turbidimetric tube and preheated at 100 r/min for 3 min on the whole-blood aggregator, and 500 µL of adenosine diphosphate (10 µmol/L) was added for a 5 min reaction. The maximum aggregation rate (MAR) of platelet aggregation was measured. The platelet aggregation ability was evaluated by reference to the MAR [18, 19]. The inhibition of platelet aggregation (IPA) in the patient was calculated as follows.

2 $$ IPA=\left({MAR}_{0}-{MAR}_{8}\right)\times 100\text{\%}$$

*MAR*_0_ and *MAR*_8_ represent the MARs corresponding to platelet aggregation before and 8 h after medication, respectively.

A thromboelastogram was used to detect the antiplatelet activity of clopidogrel. The method in reference [20] was adopted to evaluate the antiplatelet activity of clopidogrel by using the ADP inhibition rate, and an ADP inhibition rate < 10% was considered resistant to clopidogrel. The inhibition rate of ADP by clopidogrel is low when it ranges from 10 to 30%, and an inhibition rate of DP ≥ 30% is good with clopidogrel.

### Statistical methods

SPSS 22.0 was used. The means ± standard deviations ($\overline{\text{x}}$± s) of the normally distributed measurement data were expressed, and the data were normally distributed and had uniform variance. Independent sample *T* tests were used to compare variables between two groups, and variance analysis was used to compare measurement data among multiple groups. The count data were indicated as percentages (%) and were analyzed using the chi-square test. A *P* value < 0.05 indicated that the difference was statistically significant.

## Results

### Clopidogrel Lc-MS-MS method of validation

**Fig. 2:**
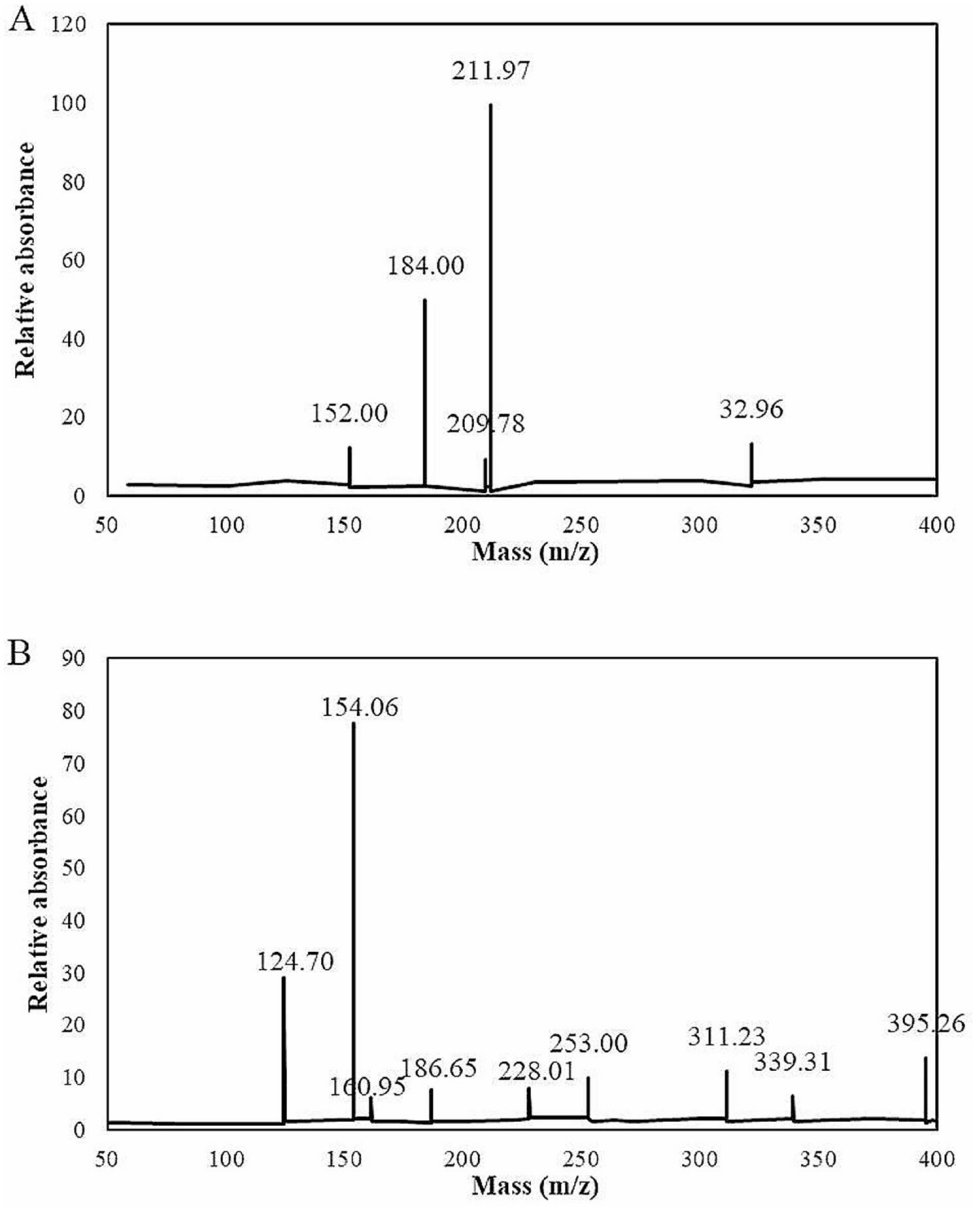
Ion mass spectrometry (MS) of clopidogrel and ticlopidine products. (A: clopidogrel; B: ticlopidine)

Product particle scanning analysis of clopidogrel and its internal standard ticlopidine was performed by electron spray ionization (Fig. 2). The maximum absorbance of clopidogrel was found at m/z 211.97, and the corresponding relative absorbance was 99.50. The maximum relative absorbance of ticlopidine at m/z 154.06 was 77.69.

**Fig. 3:**
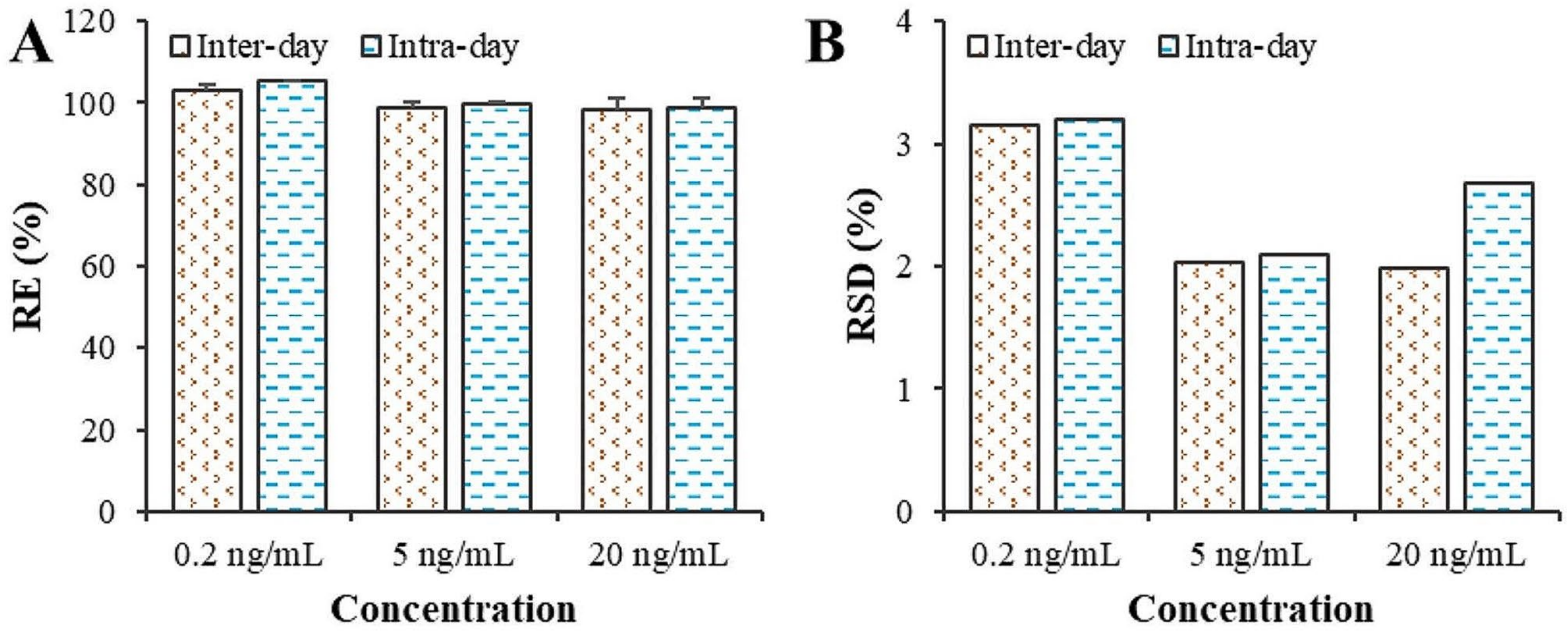
The accuracy and precision of the LC‒MS/MS method

The statistical results of the intraday and interday accuracy (RE) and precision (RSD) of clopidogrel detection using the LC‒MS/MS method are illustrated in Fig. 3. The intraday and interday accuracies (REs) of 0.2 ng/mL clopidogrel were 103.2 ± 1.05% and 105.41 ± 0.03%, respectively, falling within the range of 80–120%. For 5 ng/mL and 20 ng/mL clopidogrel, the intraday and interday accuracies (REs) were within the range of 85–125%, indicating the high accuracy of clopidogrel detection via the LC‒MS/MS method. The intraday and interday RSDs of 0.2 ng/mL clopidogrel were 3.15% and 3.20%, respectively, both of which were less than 20%. For 5 ng/mL clopidogrel, the intraday and interday RSDs were 2.03% and 2.09%, respectively, while for 20 ng/mL clopidogrel, they were 1.98% and 2.68%, respectively. The intraday and interday RSDs for 5 ng/mL and 20 ng/mL clopidogrel were less than 15%, indicating high precision in clopidogrel detection via the LC‒MS/MS method.

**Fig. 4:**
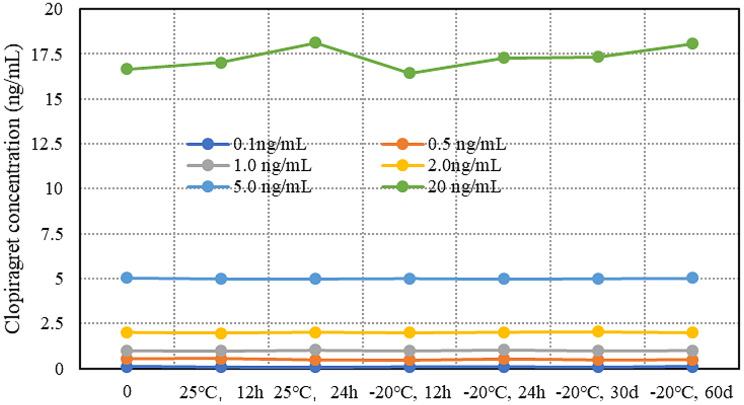
Clopidogrel stability test curve

Concentrations of 0.1 ng/mL, 0.5 ng/mL, 1.0 ng/mL, 2.0 ng/mL, 5.0 ng/mL, and 20 ng/mL clopidogrel were tested again after storage for different durations to verify their stability. The results are shown in Fig. 4. Under different conditions and storage times, the stability of clopidogrel at different concentrations was relatively ideal.

### Analysis of the CYP2C19 gene detection results

**Fig. 5:**
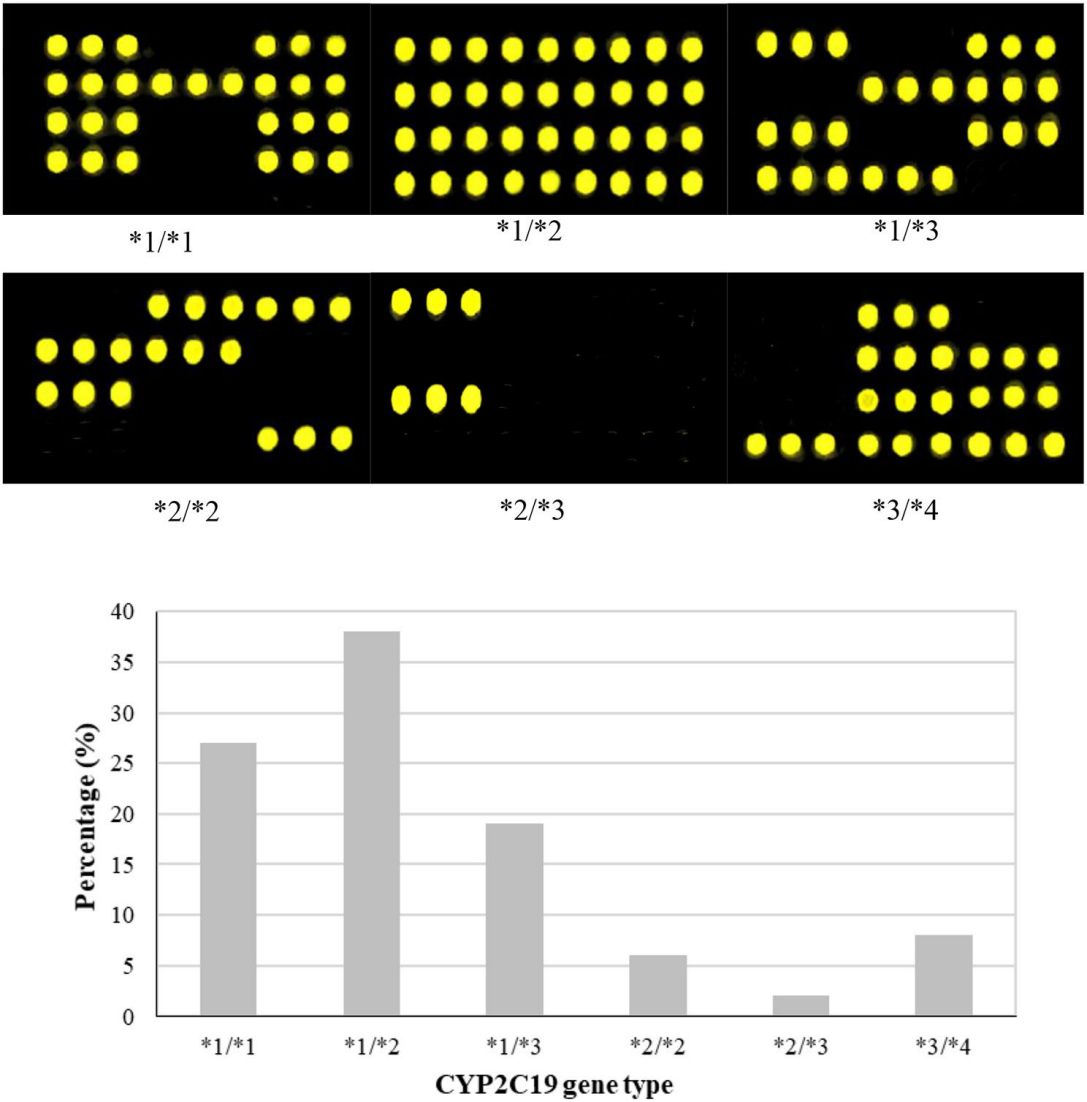
CYP2C19 genotyping results

Genotyping of the CYP2C19 gene of the included subjects was performed by gene chip technology. Figure 5 shows that the CYP2C19 gene was detected in mainly the CYP2C19*1/*1, CYP2C19*1/*2, CYP2C19*1/*3, CYP2C19*2/*2, CYP2C19*2/*3, and CYP2C19*3/*4 strains. Statistical analysis of patients with different CYP2C19 gene types was also performed. There were 38 patients (38%) with the CYP2C19*1/*2 phenotype, followed by 27 patients (27%) with the CYP2C19*1/*1 phenotype. There were 19 patients (19%) with CYP2C19*1/*3, 8 patients (8%) with CYP2C19*3/*4, 6 patients (6%) with CYP2C19*2/*2, and at least 2 patients (2%) with CYP2C19*3/*4.

### Comparison of basic data of patients in different groups

All patients were classified into the WT group (*1/*1), HTM group (CYP2C19*1/*2, CYP2C19*1/*3), or HMM group (CYP2C19*2/*2, CYP2C19*2/*3, and CYP2C19*3/*3). Of these, 27 were in the WT group, 57 were in the HTM group, and 16 were in the HMM group. In the three groups, age, sex ratio, body mass (BMI), disease course, preoperative white blood cell (WBC) count, hemoglobin (Hb) level, platelet count (PLT), and total cholesterol (TC) were compared and analyzed. The results are shown in Table 1. There were no significant differences in age; sex ratio; BMI; course of disease; or preoperative WBC, Hb, PLT, or TC among the three groups (*P* > 0.05). F represents the ratio of the amount of drug entering the systemic circulation to the dose used. P stands for pulse.

**Table 1:** Comparison of basic data between the included patients

Factor	Wild group (*n* = 27)	Heterozygous mutation group (*n* = 57)	Homozygous mutation group (*n* = 16)	χ^2^/F	P
Age (years old)	65.52 ± 8.56	64.92 ± 8.41	65.46 ± 7.74	1.105	0.338
Gender [cases (%)]				1.495	0.476
Male	18 (66.00%)	37 (66.67%)	11 (64.91%)		
Female	9 (33.33%)	20 (35.09%)	5 (31.25%)		
BMI (kg/m^2^)	24.73 ± 3.12	25.01 ± 3.09	24.97 ± 2.73	1.098	0.266
Course of disease (years)	0.61 ± 0.11	0.63 ± 0.09	0.62 ± 0.12	1.125	0.274
WBC(×10^9^/L)	7.05 ± 1.98	7.19 ± 1.87	7.10 ± 1.72	0.069	0.934
Hb (g/L)	134.68 ± 13.06	132.91 ± 13.78	135.03 ± 12.96	0.458	0.336
PLT (×10^9^/L)	185.23 ± 15.95	182.96 ± 16.97	184.87 ± 17.32	0.094	0.891
TC (mmol/L)	4.46 ± 1.02	4.26 ± 0.95	4.71 ± 0.87	0.023	0.992

### Comparison of disease histories among different groups

**Fig. 6:**
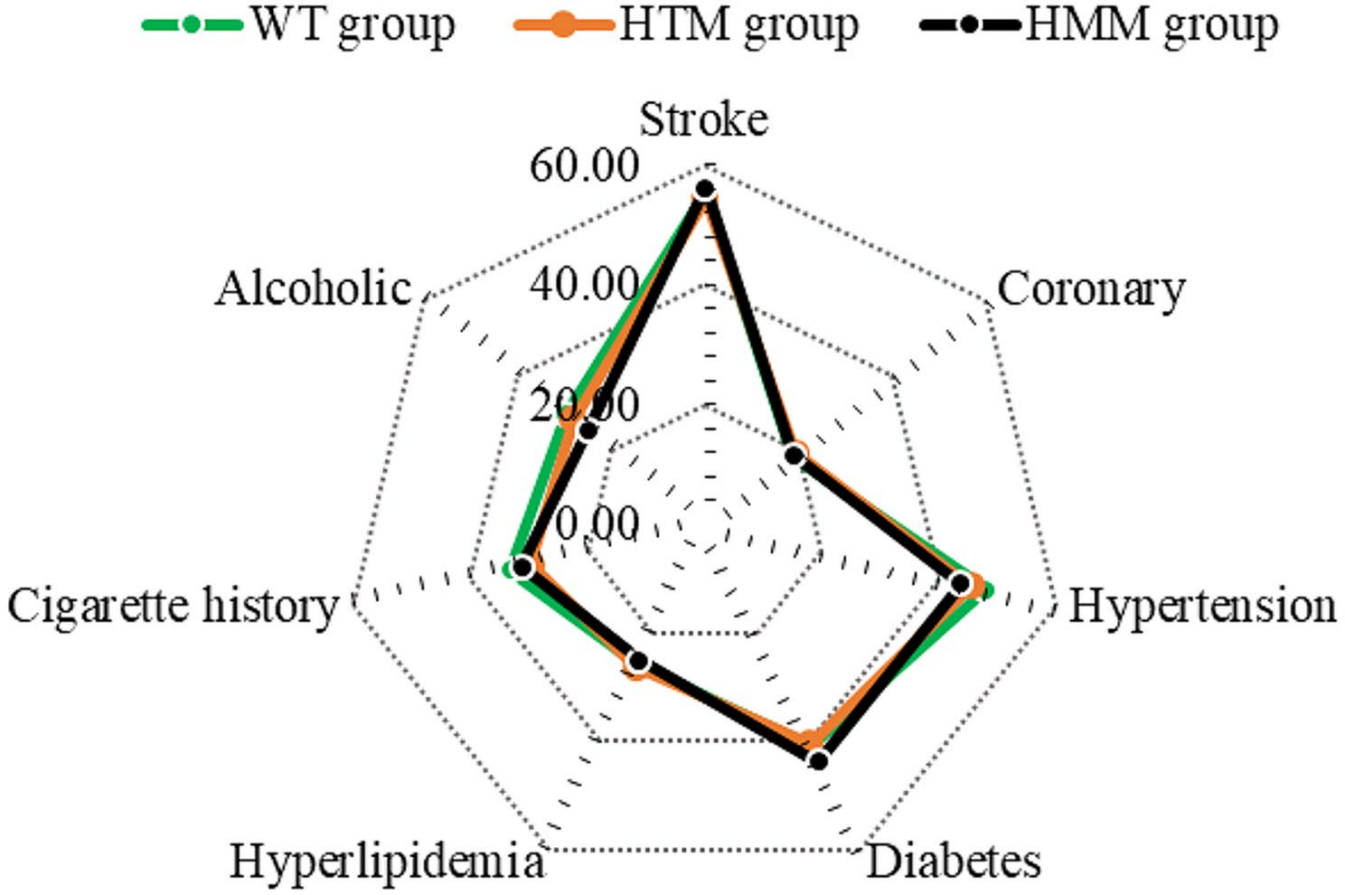
Comparison of disease history among different groups of patients

The proportions of patients with a history of stroke, hypertension, coronary heart disease, diabetes, hyperlipidemia, smoking, and alcohol consumption were compared among the three groups (Fig. 6). There were no significant differences in the proportions of patients with a history of stroke, hypertension, coronary heart disease, diabetes, hyperlipidemia, smoking, or drinking among the three groups (*P* > 0.05).

### Comparison of platelet aggregation in different groups

**Fig. 7:**
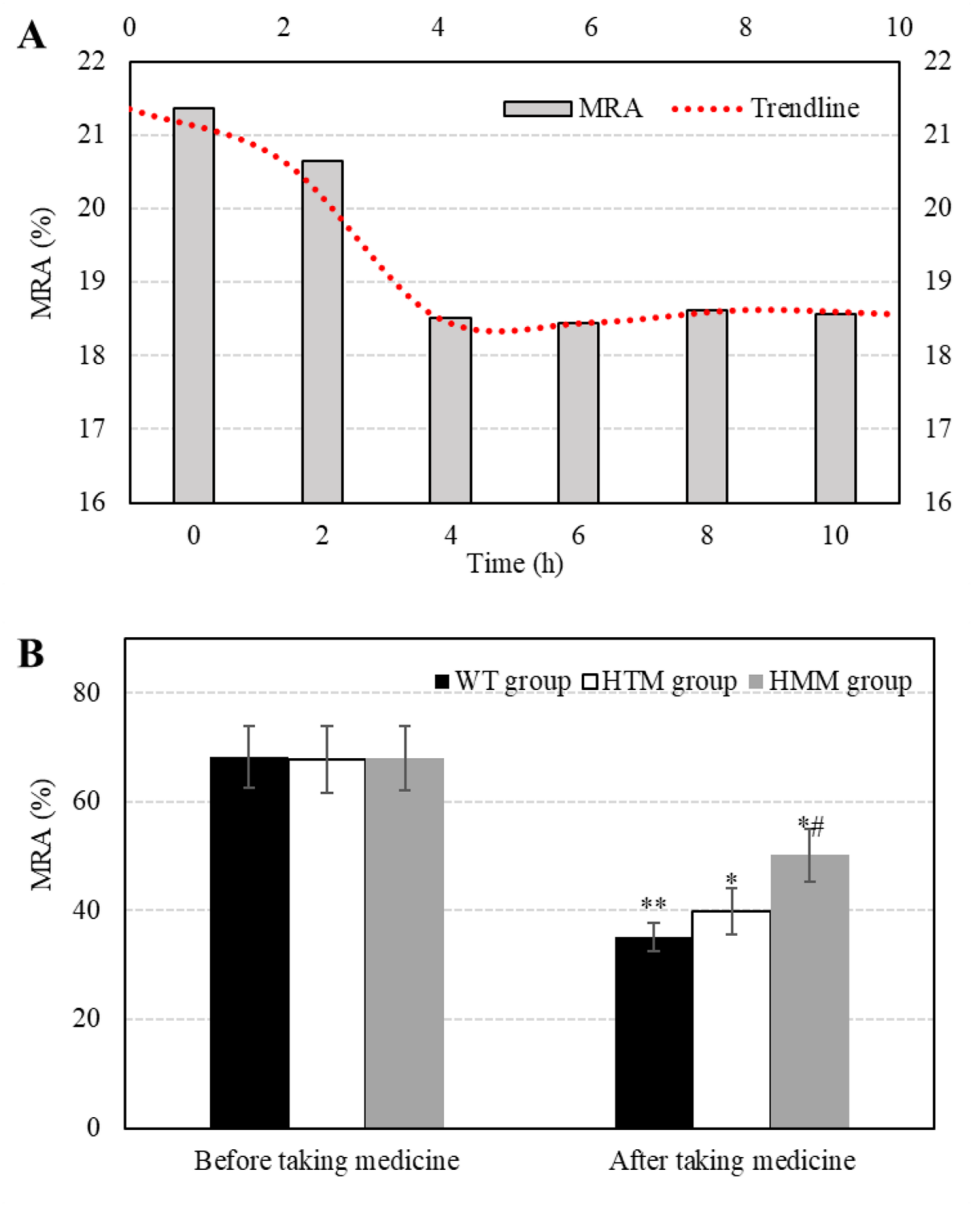
Comparison of MRA results among the different groups. (A: changes in MRA over time; B: comparison of MRA among groups; ** *P* < 0.01 vs. before medication; * *P* < 0.05 vs. before medication; # *P* < 0.05 vs. WT group.)

The platelet aggregation ability of the three groups was compared and analyzed. The indicators used to evaluate platelet aggregation ability mainly included the plasma CL, MRA, and IPA concentration and the inhibition rate of ADP. A comparison of platelet aggregation indices among the different groups is shown in Fig. 7. After clopidogrel was administered, the MRA tended to decrease first and then stabilize. In the first 4 h, the MRA showed a continuous decreasing trend, decreasing from 21.36% at 0 h to 18.52% at 4 h. With the extension of time, the MRA no longer changes, maintaining a constant state. Before treatment with clopidogrel, the MRAs of the WT, HTM, and HMM groups were 68.29 ± 5.73%, 67.79 ± 6.06%, and 68.01 ± 5.84%, respectively. There was no dramatic difference in MRA among the three groups (*P* > 0.05). After 7 days of treatment with clopidogrel, the MRAs of the WT, HTM, and HMM groups were 35.22 ± 2.61%, 50.19 ± 4.79%, and 39.87 ± 4.22%, respectively. Notably, after treatment, the MRA density in the three groups was lower than that before treatment. The MRA of the WT group was considerably different after treatment compared with before treatment (*P* < 0.01). The MRA of the HTM group and HMM group was markedly different from that before (*P* < 0.05), and the MRA in the HMM group was greatly superior to that in the WT group (*P* < 0.05).

**Fig. 8:**
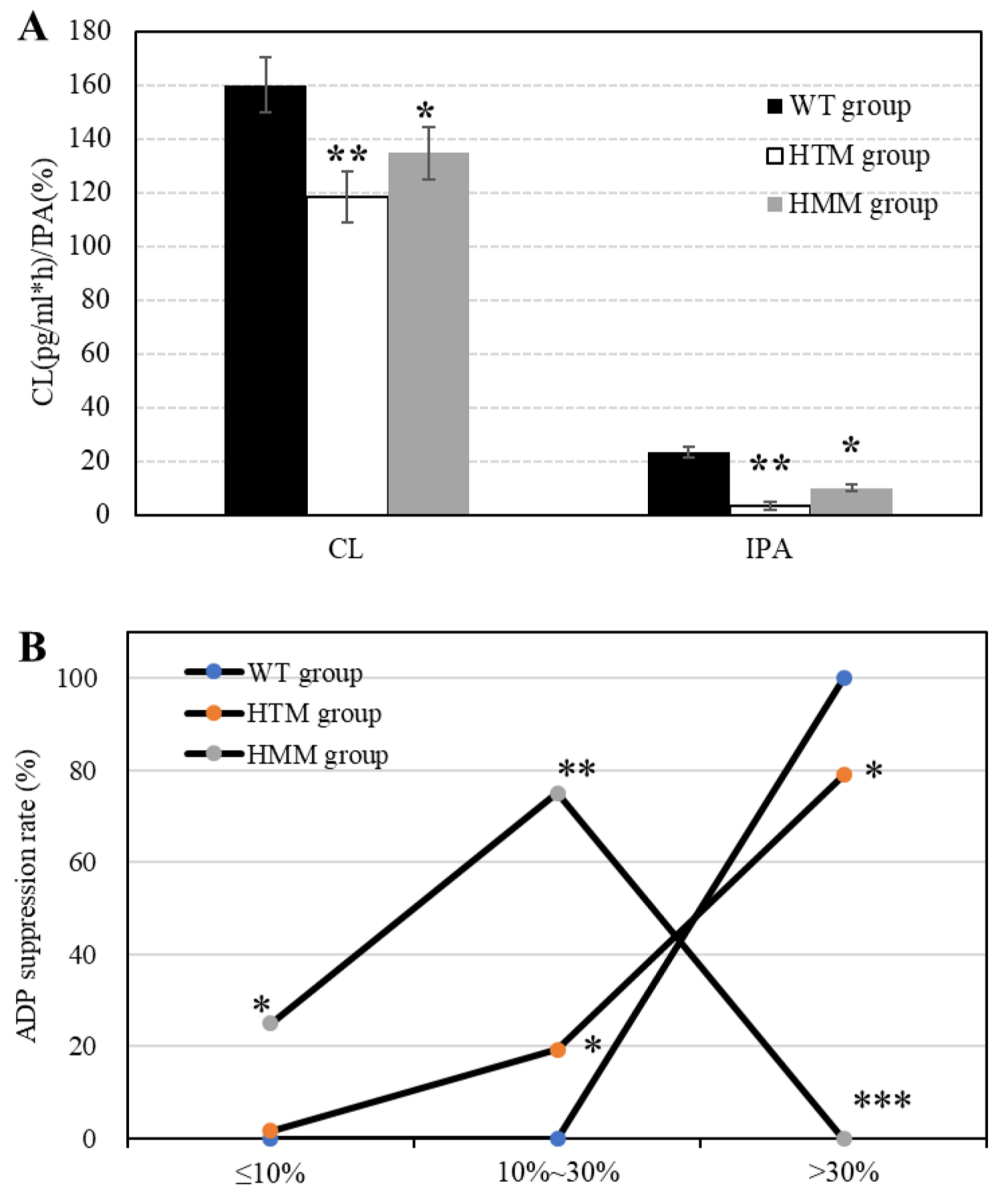
Analysis of the platelet aggregation ability of CLs and IPA and the inhibition rate of ADP. A: comparison of CL and IPA in different groups; B: comparison of the ADP inhibition rate in different groups; *** *P* < 0.001 vs. the wild-type group; ** *P* < 0.01 vs. the wild-type group; * *P* < 0.05 vs. the wild-type group

The CL, IPA, and ADP inhibition rates of patients in different groups were further compared and analyzed (Fig. 8). The CLs of the patients in the WT, HTM, and HMM groups were 160.19 ± 10.48 pg/mL*h, 118.54 ± 9.62 pg/mL*h, and 134.72 ± 9.95 pg/mL*h, respectively. CL in WT group was greatly superior to that in the HTM group (*P* < 0.01) and that in the HMM group was lower versus WT group (*P* < 0.05). The IPAs of patients in the WT, HTM, and HMM groups were 23.28 ± 1.87%, 3.28 ± 1.29%, and 10.01 ± 1.12%, respectively. The IPA of the WT patients was dramatically superior to that of the HTM patients (*P* < 0.01), and that of the HMM patients was lower than that of the WT patients (*P* < 0.05). There were 0, 1 (1.75%), and 4 (25.00%) patients with ADP inhibition rates ≤ 10% in the wild-type group, HTM group, and HMM group, respectively. There were 0, 11 (19.30%), and 12 (75.00%) patients with ADP inhibition rates of 10-30%. There were 27 patients (100%), 45 patients (78.95%), and 0 patients with ADP inhibition rates greater than 30%. Furthermore, the proportions of patients with ADP inhibition rates ≤ 10% and 10%-30% in the HTM group were dramatically different from those in the WT group (*P* < 0.05). The proportion of patients with an ADP inhibition rate ≤ 10% in the HMM group was dramatically different from that in the WT group (*P* < 0.05). The proportion of 10-30% patients was dramatically greater than that in the WT group (*P* < 0.01), and the proportion of patients older than 30% was evidently inferior to that in the WT group (*P* < 0.001).

### Comparison of reischemic events in different groups of patients after treatment

**Fig. 9:**
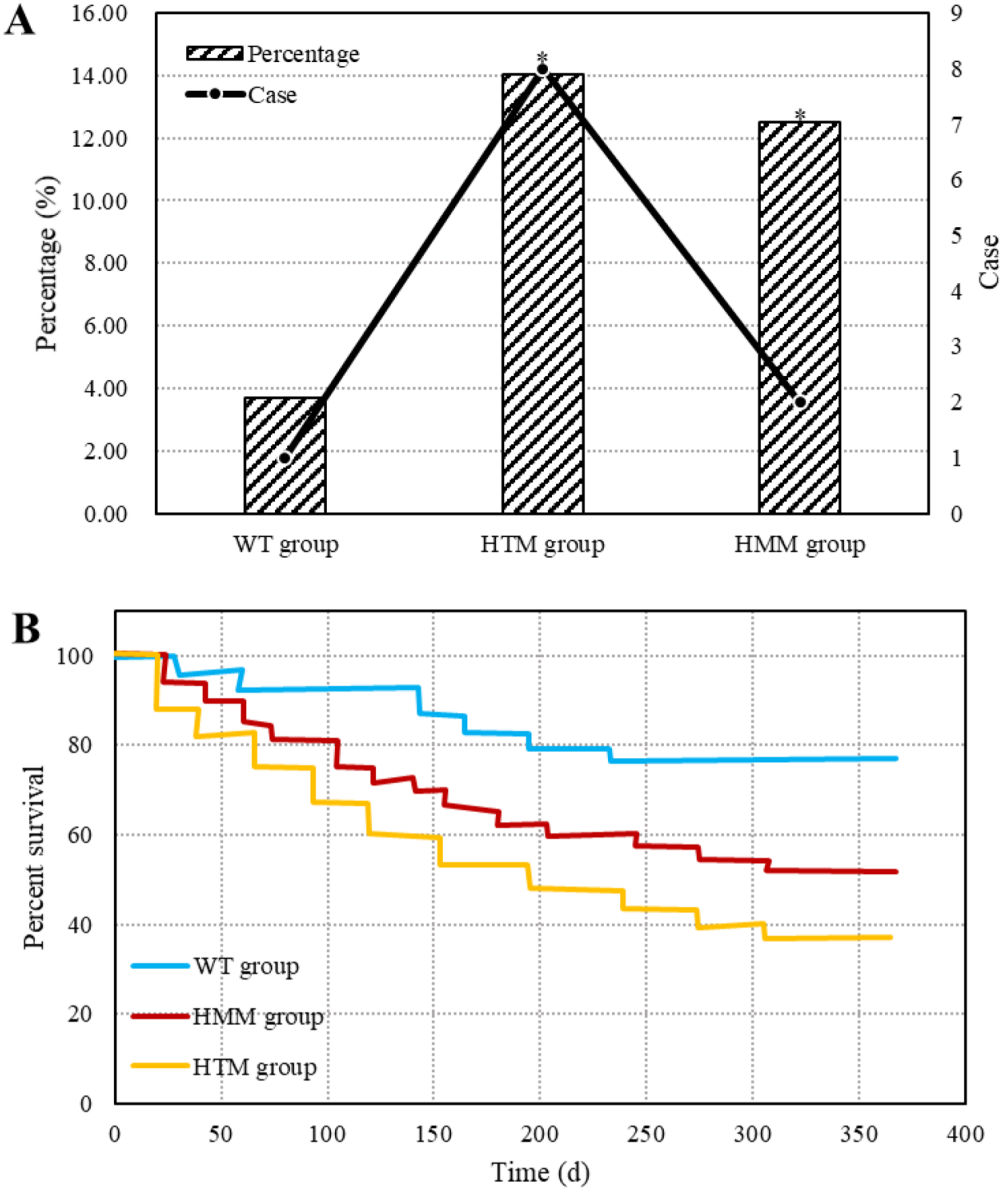
Comparison of reischemic events in different groups of patients after treatment. A: comparison of the incidence of reischemic events after treatment in different groups; B: Kaplan‒Meier curve of postoperative reischemic events in different groups; * *P* < 0.05 vs. the WT group

Patients in the three groups were followed up for 12 months, after which the incidence of postoperative reischemia events was analyzed (Fig. 9). One patient in the wild group experienced reischemic events, for an incidence of 3.70%. Eight patients (14.04%) in the HTM group developed reischemic events. Two patients (12.50%) in the HMM group experienced reischemic events. K‒M curves were plotted to analyze the reaemia events in the three groups. The results showed that patients in the HTM group had a remarkably greater rate of reischemic events during 12 months of follow-up than did those in the wild-type group (HR = 2.028, 95% CI = 1.025–4.359; *P* = 0.029). The incidence of reischemic events in the HMM group was greatly greater than that in the wild-type group within 12 months of follow-up (HR = 5.236, 95% CI = 1.179–8.352, *P* = 0.034). There was no significant difference in the incidence of reischemic events within 12 months between patients in the HTM group and those in the HMM group (HR = 0.587, 95% CI = 0.253–1.428, *P* = 0.193).

### Comparison of postoperative CR and stent thrombosis rates among different groups

**Fig. 10:**
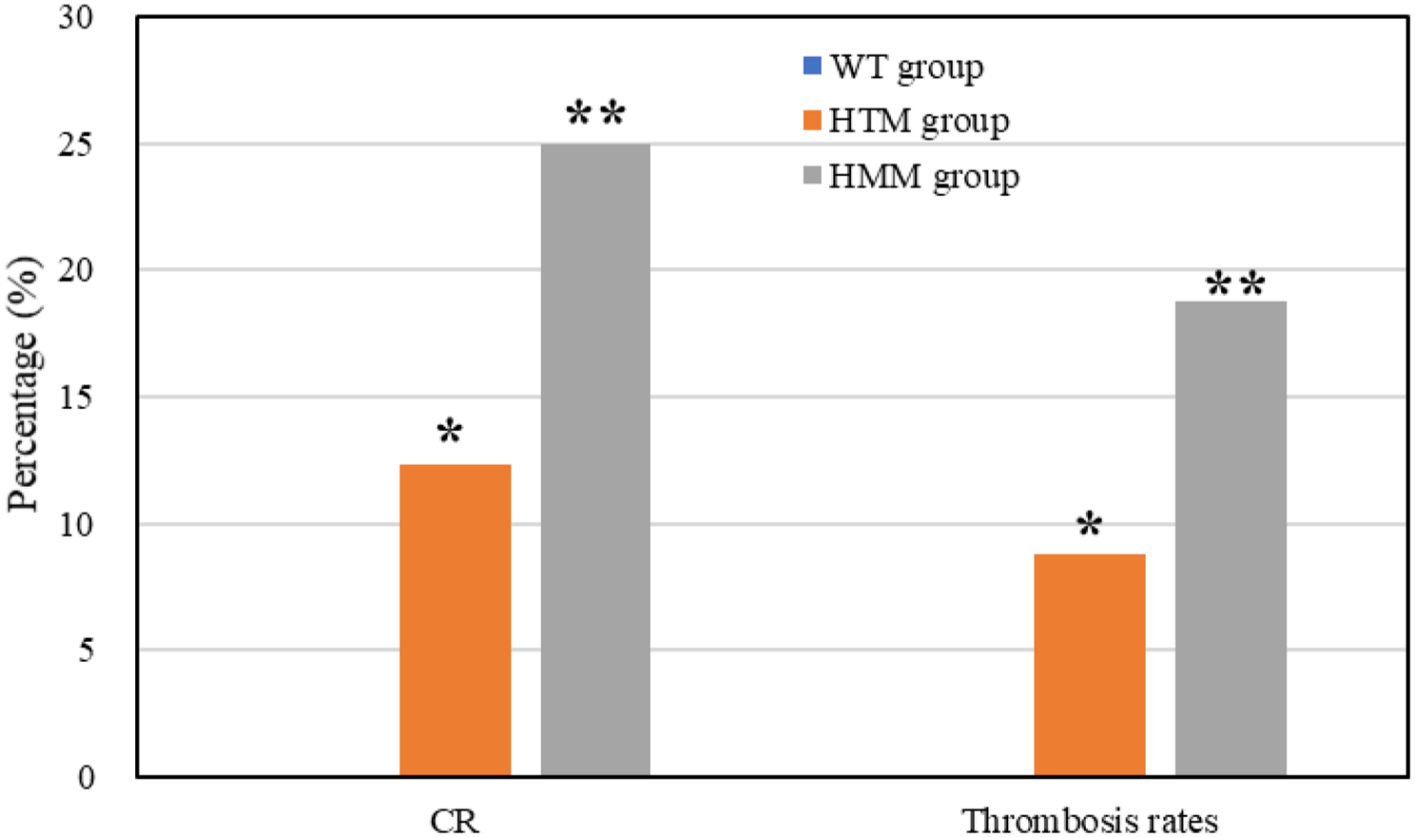
Comparison of postoperative CR and stent thrombosis rates among different groups of patients. (* *P* < 0.05 vs. the WT group. **P < 0.01 vs. the WT group.)

The postoperative CR and thrombosis rates of patients in the three groups were compared and analyzed (Fig. 10). In the WT, HTM, and HMM groups, 0, 7 (12.28%), and 4 (25.00%) patients, respectively, achieved CR. The incidences of thrombosis were 0, 5 (8.77%), and 3 (18.75%). The CR and thrombosis rates of patients in the HTM group were markedly different from those in the WT group (*P* < 0.05), and the CR and thrombosis rates of patients in the HMM group were considerably different from those of patients in the WT group (*P* < 0.01).

## Discussion

In this study, 100 patients with ischemic cerebrovascular disease requiring CAS treatment were treated with 75 mg of clopidogrel, and the pharmacokinetics, pharmacodynamics, and genetic polymorphisms of clopidogrel in their blood were analyzed. Clopidogrel is rapidly absorbed in vivo, and the plasma clopidogrel concentration decreases markedly after 2 h of treatment [21]. Several researchers noted that at least half of clopidogrel was absorbed according to its urine excretion after 2 h of administration [22]. After patients take clopidogrel, it is rapidly transformed into carboxylate derivatives after liver metabolism, so the concentration of clopidogrel in blood is low [23]. HPLC‒MS-MS was used to detect clopidogrel in blood. HPLC‒MS-MS has the advantages of high sensitivity and good specificity and has dramatic advantages in the detection of low-concentration samples. The results showed that the maximum absorbance of clopidogrel was at m/z 211.97, and the corresponding relative absorbance was 99.50. The maximum relative absorbance of ticlopidine at m/z 154.06 was 77.69. The stability of clopidogrel at different concentrations was better under different conditions and for different storage times. These results indicated that HPLC‒MS-MS was feasible for detecting plasma drug concentrations, and plasma samples were stable at room temperature and frozen after treatment; thus, HPLC‒MS-MS could be used in the clinical monitoring of plasma drug concentrations and pharmacokinetics.

In recent years, the incidence of atherosclerosis has increased markedly in China. The progression of this disease can lead to various cardiovascular and cerebrovascular diseases. According to statistics, stroke caused by atherosclerosis accounts for 1/5 of ischemic stroke cases [24], and stroke is one of the main causes of death worldwide. Currently, the clinical treatment methods for atherosclerosis mainly include drug therapy and CAS [25]. CAS is characterized by less trauma and faster postoperative recovery than other surgical methods; therefore, CAS is currently the main clinical treatment for atherosclerosis [26]. However, there is still a risk of vascular restenosis after CAS, so patients still need to take long-term antiplatelet aggregation drugs for prevention after treatment [27]. Although antiplatelet aggregation drugs can effectively reduce the incidence of in-stent restenosis, 5%∼20% of in-stent restenosis adverse events still occur [28]. Clopidogrel is a commonly used drug in antiplatelet therapy after CAS because of its advantages in antiplatelet therapy [29]. Clopidogrel is a thiophenpyridine precursor drug. Clopidogrel itself has no effect. After patients take clopidogrel, it is transformed into an active substance with antiplatelet aggregation after the action of the CYP450 enzyme system in vivo. As a member of the cytochrome P450 family, CYP2C19 plays an important role in drug metabolism. The CYP2C19 gene is polymorphic, and the wild-type CYP2C19*1/*1 gene can promote the conversion of clopidogrel into active components. However, the deletion of the CYP2C19*1/*2, CYP2C19*1/*3, CYP2C19*2/*2, CYP2C19*2/*3, and CYP2C19*3/*3 genotypes markedly reduced the amount of clopidogrel active ingredients released. Thus, the antiplatelet effect of clopidogrel is weakened [30]. There were 38 patients (38%) with the CYP2C19*1/*2 phenotype, followed by 27 patients (27%) with the CYP2C19*1/*1 phenotype. There were 19 patients (19%) with CYP2C19*1/*3, 8 patients (8%) with CYP2C19*3/*4, 6 patients (6%) with CYP2C19*2/*2, and at least 2 patients (2%) with CYP2C19*3/*4. Among the included patients with ischemic cerebrovascular disease, the CYP2C19*1/*2 phenotype was the most common, and only 27% of patients with a normal CYP2C19 genotype were included. Hence, CYP2C19 gene mutations of different degrees are present in most patients. Some studies showed that the proportion of the wild-type CYP2C19 gene population in China is less than 40%, while that of Caucasians can reach 70% [31]. The results showed that, similar to current domestic statistical results, only 27% of patients with the wild-type CYP2C19 gene were infected [32]. Morales-Rosado et al. (2021) [33] noted that the CYP2C19*2 and CYP2C19*3 genotypes account for approximately 27% and 5%, respectively, of the Chinese population, and that the number of patients with impaired clopidogrel metabolism caused by the CYP2C19 gene mutation is approximately 15-17%. In this study, 16% (16/100) of the CYP2C19*2 and CYP2C19*3 genotypes were mutated, which was consistent with the current domestic statistical results. Therefore, the efficacy of clopidogrel in the domestic population may differ greatly in the later stage.

MRA can reflect platelet activity in vivo and is a common indicator for evaluating platelet aggregation function and can indirectly reflect the anticoagulant effect of clopidogrel [34]. Turbidimetry, as a commonly used method for detecting platelet aggregation rates, has the advantages of simple operation, short detection time, and affordability and has dramatic advantages in detecting platelet aggregation rates [35]. Turbidimetric analysis was performed to detect and analyze MRAs in different groups of patients. The results showed that the MRA decreased first and then stabilized after patients took clopidogrel, and in the first 4 h, the MRA percentage continued to decrease from 21.36% at 0 h to 18.52% at 4 h. Then, as time progressed, the MRA no longer changed, maintaining a constant state. MRA decreased markedly after treatment with clopidogrel compared with before treatment in the three groups. The MRA of the WT group was considerably different after treatment compared with before treatment (*P* < 0.01). The MRA of the HTM group and HMM group was markedly different from that before (*P* < 0.05), and the MRA of the HMM group was dramatically greater than that of the WT group (*P* < 0.05). The results showed that more than 80% of the patients had been treated with effective active substances within 4 h after taking clopidogrel. After clopidogrel was administered, the platelet aggregation rate of the patients decreased to varying degrees, and the platelet aggregation rate of the wild-type group declined the fastest. The second group was the HTM group, and the HMM group had the slowest decrease in the platelet aggregation rate. The rate of MRA decrease in the CYP2C19 HMM group was notably lower than that in the WT group. It was suggested that CYP2C19 has a dramatic impact on the efficacy and function of clopidogrel, which is similar to the results of other works [36]. As an adenosine diphosphate receptor antagonist, clopidogrel can specifically bind to ADP receptors on platelet membranes [37], and the combination of clopidogrel and ADP can inhibit platelet aggregation caused by high concentrations of adenosine diphosphate [38]. The results showed that the proportions of patients in the HTM group with ADP inhibition rates ≤ 10% and 10%-30% were markedly different from those in the WT group (*P* < 0.05). The proportion of patients with an ADP inhibition rate ≤ 10% in the HMM group was dramatically different from that in the WT group (*P* < 0.05). The proportion of 10%∼30% patients was dramatically greater than that in the WT group (*P* < 0.01), and the proportion of patients older than 30% was evidently inferior to that in the WT group (*P* < 0.001). It was suggested that the ADP inhibition rate of patients with ischemic cerebrovascular disease caused by a CYP2C19 gene mutation decreases markedly after taking clopidogrel, increasing the incidence of cardiovascular events [39]. The results showed that patients in the HTM group had a remarkably greater rate of reischemic events during 12 months of follow-up than did those in the wild-type group (HR = 2.028, 95% CI = 1.025–4.359, *P* = 0.029). The incidence of reischemic events in the HMM group was greatly greater than that in the wild-type group within 12 months of follow-up (HR = 5.236, 95% CI = 1.179–8.352, *P* = 0.034). There was no significant difference in the incidence of reischemic events within 12 months between patients in the HTM group and those in the HMM group (HR = 0.587, 95% CI = 0.253–1.428; *P* = 0.193). Davis et al. (2020) [40] showed that the clinical outcome of the CYP2C19 heterozygous genotype was better than that of the CYP2C19 homozygous genotype in patients with acute myocardial infarction, and the results in this work were similar to those of the CYP2C19 heterozygous genotype. The CR and thrombosis rates of patients in the HTM group were markedly different from those in the WT group (*P* < 0.05), and the CR and thrombosis rates of patients in the HMM group were considerably different from those of patients in the WT group (*P* < 0.01). These results suggested that CR and thrombosis rates are considerably different among CYP2C19 patients with different genotypes. Chi and Wang (2019) [41] noted that the CYP2C19 gene mutation is a risk factor for postoperative thrombosis after CAS, and the homozygous type is strongly correlated with postoperative thrombosis after CAS.

## Conclusions

The effect of the CYP2C19 gene polymorphism on the antiplatelet efficacy of clopidogrel after CAS was analyzed based on network pharmacology. The results showed that the CYP2C19 gene affected CR occurrence and stent thrombosis after CAS by influencing clopidogrel metabolism and the platelet aggregation rate, thus affecting patient prognosis. However, there are still several limitations in this study. The duration of blood collection is limited, and factors such as patient physiological status can affect clopidogrel drug metabolism and efficacy. In the future, the time point of blood collection will be further optimized, and the effects of physiological conditions on clopidogrel metabolism and efficacy will be considered to further study the effects of clopidogrel on platelet aggregation. In conclusion, this study provides a clinical reference for the formulation of individualized clinical medication regimens.

## Data Availability

The experimental data used to support the findings of this study are available from the corresponding author upon request.
